# The alkali degradation of LDPE-based radiation-grafted anion-exchange membranes studied using different e*x situ* methods†

**DOI:** 10.1039/d0ra06484j

**Published:** 2020-10-05

**Authors:** Kelly M. Meek, Carly M. Reed, Bryan Pivovar, Klaus-Dieter Kreuer, John R. Varcoe, Rachida Bance-Soualhi

**Affiliations:** Department of Chemistry, School of Chemistry and Chemical Engineering, University of Surrey Guildford GU2 7XH UK j.varcoe@surrey.ac.uk; Electrochemical Engineering & Materials Chemistry, National Renewable Energy Laboratory (NREL) Golden Colorado 80401 USA; Max-Planck-Institut für Festkörperforschung (MPI-FKF) Heisenbergstrasse 1 D-70569 Stuttgart Germany; Department of Chemical and Biological Engineering, University of Ottawa 161 Louis Pasteur Ottawa Ontario K1N 6N5 Canada

## Abstract

Radiation-grafted anion-exchange membranes (RG-AEM) in alkaline membrane fuel cells (AEMFC) exhibit promising performances (low *in situ* resistances, high power outputs and reasonably high alkali stabilities). Much research is focused on developing AEMs with enhanced chemical stabilities in the OH^−^-forms at temperatures >60 °C. This study contributes towards this effort by providing a comparison of three different *ex situ* methods of screening alkali stabilities (where different laboratories conducted experiments on exactly the same batches of RG-AEM). Vinylbenzyl chloride monomer was radiation-grafted onto 25 μm thick low-density polyethylene (LDPE) precursor film in a single batch. This batch of grafted membrane was then split into three sub-batches, which were converted into RG-AEMs *via* amination with either: trimethylamine (TMA), *N*-methylpyrrolidine (MPY), or *N*-methylpiperidine (MPIP). Samples of each RG-AEM (l-AEM-TMA, l-AEM-MPY, and l-AEM-MPIP) were then distributed between the three collaborating institutes for evaluation using each institutes' test protocols. Out of the three head-group chemistries, the l-AEM-TMA generally exhibits the best balance of conductivity and *ex situ* alkali degradation, especially in lower humidity environments. The l-AEM-TMA also exhibited interestingly high Cl^−^ ion conductivities (*ca.* 100 mS cm^−1^) when heated to 80 °C in a relative humidity RH = 95% atmosphere, a measurement frequently overlooked in favour of determining conductivities of RG-AEMs submerged in water (conductivities of submerged RG-AEMs can be suppressed due to excessive water contents and swelling).

## Abstract 2

Three different *ex situ* alkali degradation protocols were compared on single batches of LDPE-based radiation-grafted anion-exchange membranes (containing trimethylammonium, *N*-methylpiperidinium, and *N*-methylpyrrolidinium headgroups).

## Graphical abstract

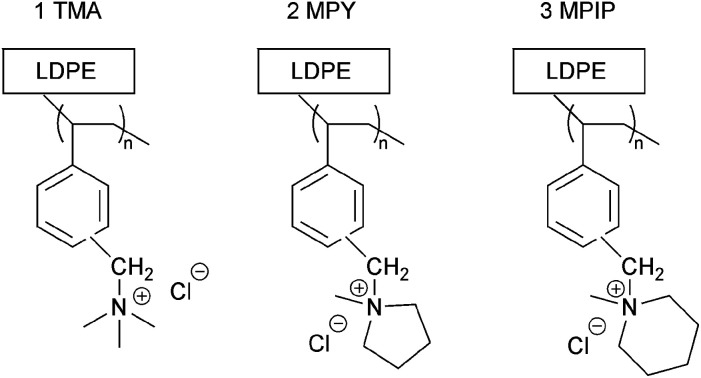

## Introduction

Anion-exchange membranes (AEM) are being developed for a variety of electrochemical technologies.^1^ Alkali stable types are required for many applications, including alkaline membrane fuel cells (AEMFC) and AEM-based electrolysers.^2–19^ A high performance class of AEM is made *via* radiation-grafting (RG-AEM),^3,20–28^ with examples being published as far back as 1996.^29^ Poly(ethylene-*co*-tetrafluoroethylene)-(ETFE)-based RG-AEMs have been the most commonly investigated, with those made using trimethylamine (TMA), *N*-methylpyrrolidine (MPY), and *N*-methylpiperidine (MPIP) amination agents having good stabilities in aqueous alkali.^30^ RG-AEMs have been tested in AEMFCs alongside ETFE-based RG anion-exchange ionomer (RG-AEI) powders in the electrodes.^3,11,31^

In 2017, it was shown that the RG process (involving a high dose-rate electron-beam pre-irradiation peroxidation method) was improved when the organic solvent (propan-2-ol), used in the vinylbenzyl chloride (VBC) monomer-containing grafting mixture, was replaced with water.^32^ This development then enabled the fabrication of RG-AEMs using low-density polyethylene (LDPE) as a substrate, rather than partially-fluorinated ETFE (the radiation grafting of VBC onto LDPE did not work with propan-2-ol-based grafting mixtures).^33^ While the use of ETFE-based RG-AEMs was subject to a temperature limit of 60 °C (due to mechanical fragility above this temperature),^33^ LDPE-based RG-AEMs retained their mechanical integrity inside AEMFCs when in the OH^−^-form at >60 °C, allowing for the testing of RG-AEMs in AEMFCs to be routinely conducted at 80 °C.^34,35^

This previously developed LDPE-based class of RG-AEM^33,34^ was selected for use in this study, to provide a comparison of select *ex situ* methods for evaluating the alkali stability of AEMs. As RG-AEMs made with TMA, MPY, and MPIP amination agents have some of the best alkali stabilities reported,^30^ we investigated LDPE-based RG-AEMs containing these three head-group chemistries (designated as l-AEM-X, where X is the amine used in the fabrication). Note, other olefins (*e.g.* HDPE) can be used,^11^ but optimisation work is ongoing to allow production of large batches with reproducible properties.

Historically, different labs have used a diversity of *ex situ* methods for determining the alkali stabilities of AEMs. Therefore, the aim of this study was to conduct a comparison of three different *ex situ* methods with different characteristics:^36^

### (1) Heating the RG-AEMs in aqueous alkali

The conditions experienced by the AEMs in this type of test do not relate to those *in situ* (the AEM is not submerged in aqueous electrolyte containing spectator cations and anions in a fuel cell environment). Despite this, it is a useful way of quickly screening out AEMs with poor alkali stabilities and it is the most common method found in the literature, hence we included it in our study.

### (2) Heating OH^−^-form RG-AEMs in relative humidity RH = 95% atmospheres^37^

This is a high hydration alkali stability test with the absence of metal cations and excess OH^−^ anions. As such it is more relevant (*cf.* test 1 above) to the *in situ* AEMFC conditions, especially at low current densities and close to the anode (where H_2_O is electrochemically generated in the alkali hydrogen oxidation reaction). Note, the specific method adopted is more novel and was developed by the MPI-FKF labs for this study.

### (3) A lower RH thermogravimetric (TGA) method^38^

This previously published and validated method is a lower hydration alkali stability test, again without the presence of metal cations or excess OH^−^ anions. This test is more relevant to *in situ* conditions close to the cathode with AEMFCs operating at higher current densities.

## Methodology

### Synthesis of the LDPE-based RG-AEMs (l-AEM)

**Scheme 1:**
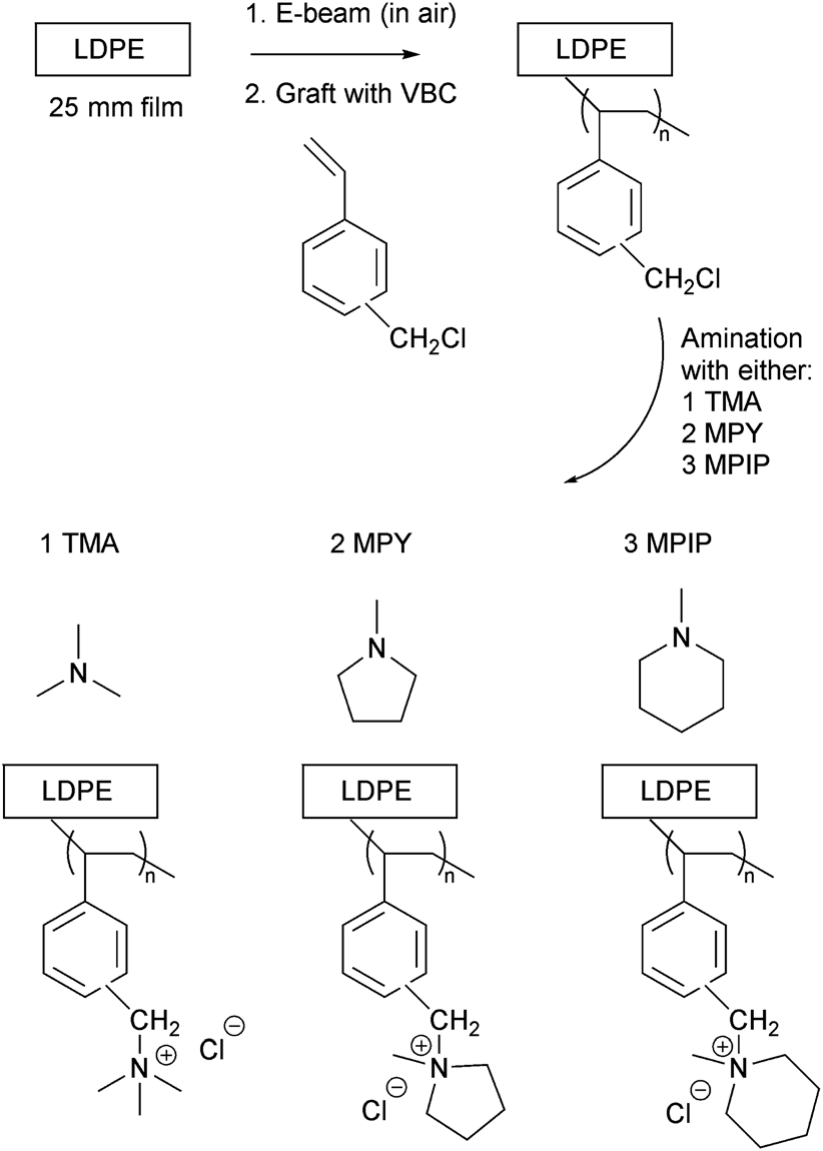
An outline of the synthesis of the LDPE-based radiation-grafted anion-exchange membranes (l-AEM-X) with the three cationic head-groups under test.

The l-AEMs were synthesized as previously reported in detail (Scheme 1).^34^ To summarize, LDPE films (25 μm thick, Goodfellow ET311126) were irradiated in air with a 4.5 MeV electron-beam to an absorbed dose of 100 kGy (Steris, Swindon, UK) and the irradiated films were then stored at −40 °C until use. For grafting, the irradiated LDPE films were submerged into a N_2_-purged (30 min) aqueous grafting mixture containing 5 wt% vinylbenzyl chloride (VBC, 3/4-isomer mix, Sigma-Aldrich product 338729, no removal of inhibitors) and 1 wt% 1-octyl-2-pyrrolidone dispersant. We do not remove inhibitors from the VBC monomer as we have found that this leads to high levels of homopolymer formation, which is hard to remove from the desired RG-AEMs. This has also been discussed previously by others.^39^ After further purging with N_2_ for 2 h, the grafting mixture containing the films was heated to 55 °C for 16 h. After grafting, the resulting membranes (designated l-poly(VBC)) were thoroughly washed in toluene, heated in toluene for 70 °C for at least 4 h, dried in a vacuum oven at 50 °C for no less than 3 h, and weighed. The degree of grafting (DoG, a standard measure of grafting yield)^40,41^ were calculated using eqn (1) to be 121%:1
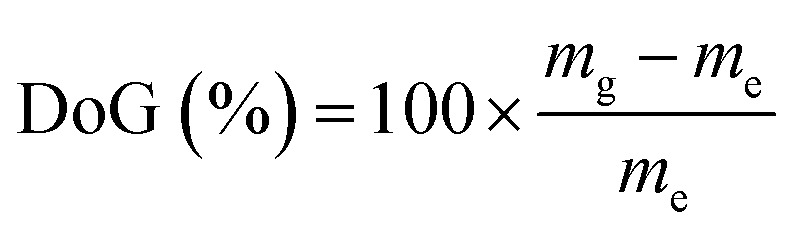
where *m*_g_ is the mass of the grafted membrane and *m*_e_ is the mass of the e^−^-beamed pre-grafted substrate films.

The l-poly(VBC) membranes were then separated into three batches and each batch was aminated with one of the following amines (to form the l-AEMs, exact amination conditions given in Table 1): TMA (aq, 50 wt%), MPY (97%), and MPIP (99%) (all from Sigma-Aldrich). After amination, the l-AEMs were subjected to multiple washings with ultrapure water (UPW, 18.2 MΩ cm resistivity) and then heated in fresh UPW for 1 h at 60 °C. The l-AEMs were then immersed in aqueous NaCl (1 mol dm^−3^) for 15 h with an exchange of this solution during this time. The final Cl^−^-form l-AEMs were obtained after multiple washings in UPW to remove any excess Na^+^ and Cl^−^ ions. The l-AEMs were stored in UPW in plastic pots until use or shipping. The l-AEMs were shipped from Surrey to the international partners (NREL and MPI-FKF) in sealed plastic bags containing a trace of water to help maintain hydration.

**Table 1:** The amination conditions used to synthesise the l-AEM-X (X = amine used in synthesis)

	l-AEM-TMA	l-AEM-MPY	l-AEM-MPIP
Amine	Trimethylamine	*N*-Methyl-pyrrolidine	*N*-Methyl-piperidine
Amine (aq) concentration	50 wt%	15 wt%	15 wt%
Amination temperature	Room temp.^a^	60 °C	60 °C
Amination time	24 h	16 h	16 h

a Heating not required. Do not heat aqueous TMA in sealed vessels to avoid the danger of pressure-induced glassware explosion.

### Raman spectroscopy on the Cl^−^-form l-AEMs

Raman spectra were recorded on each l-AEM and the l-poly(VBC) grafted intermediate membrane using a ThermoFisher DXR Raman microscope (50× objective lens, *λ* = 532 nm laser (10 mW), *ca.* <2 μm laser spot size). This was to confirm l-AEM synthesis and prove the presence of each different ammonium group.^30^ Each spectrum was recorded over the spectral range 300–3500 cm^−1^ range, as an average of 36 accumulations (2 s exposure times).

### The *ex situ* characterization of the Cl^−^-form l-AEMs (Surrey protocols)^32,33^

These initial characterisations were conducted on the Cl^−^-forms to measure the initial properties of the l-AEMs before any high pH exposure (that risks degradation). The ion-exchange capacities (IEC, mmol g^−1^), gravimetric water uptakes WU (%), thickness increases on hydration (through-plane swelling – TPS) (%), and *λ*(H_2_O) values (number of H_2_O molecules per Cl^−^ anion) were measured using the protocols previously described in detail^32,33^ and are defined below:2
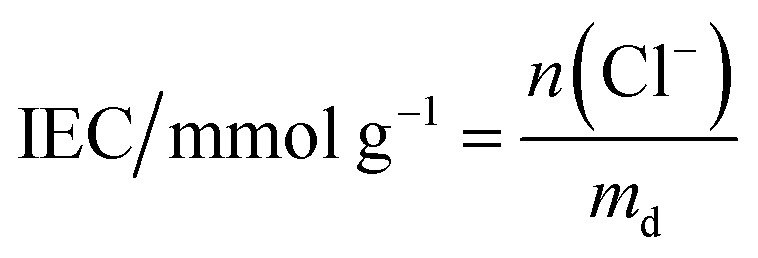
3
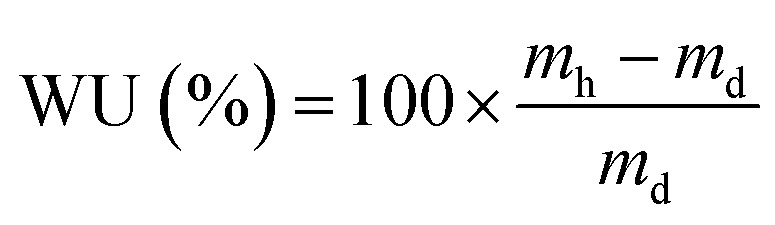
4
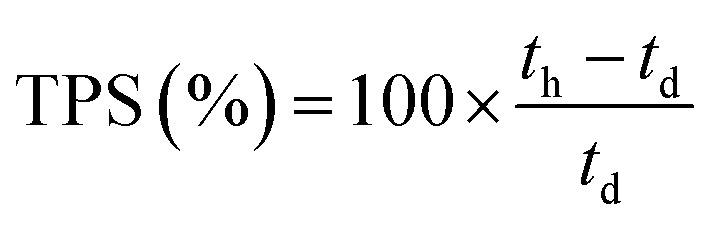
5
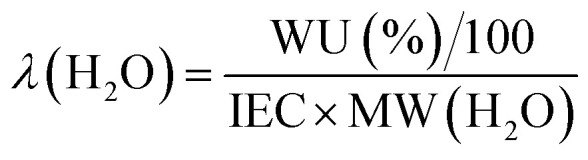
where *n* = amount/mol, *m* = mass/g, *t* = thickness/μm, MW(H_2_O) = 18.015 g mol^−1^, subscript d = dehydrated, and subscript h = hydrated. These data are summarized in Table 2 in the results section. Note that IEC is an important measure of the ionic content of ion-exchange membranes including radiation-grafted types.^40,41^

**Table 2:** A summary of the key *ex situ* properties^31^ of the Cl^−^-form l-AEMs determined in the lab at Surrey (errors are sample standard deviations from measurements on *n* = 3 samples of each l-AEM) and compared to NREL data [] and MPI-FKF mean IEC data {} to give an indication of inter-laboratory variations

Property	l-AEM-TMA	l-AEM-MPY	l-AEM-MPIP
IEC (using Cl^−^ masses)/mmol g^−1^	2.68 ± 0.15 [2.48 ± 0.01] {2.72}	2.40 ± 0.01 [2.30 ± 0.07] {2.45}	2.35 ± 0.07 [2.26 ± 0.04] {2.43}
Thickness (dry)/μm	43 ± 3 [46 ± 2]	53 ± 4 [63 ± 2]	55 ± 8 [53 ± 2]
Thickness (hydrated)/μm	54 ± 2 [54 ± 2]	62 ± 2 [65 ± 2]	66 ± 3 [66 ± 2]
TPS (%)	26 ± 6^a^ [17 ± 6^a^]	18 ± 10^a^ [3 ± 5^a^]	20 ± 17^a^ [25 ± 5^a^]
WU (%)	108 ± 7 [95^b^]	149 ± 5 [130^b^]	187 ± 26 [172^b^]
*λ*(H_2_O)	23 ± 2^a^ [21^b^]	35 ± 1^a^ [31^b^]	44 ± 6^a^ [42^b^]
Length swelling in *x*-axis direction (%)	[14]^b^^,^^c^	[18]^b^^,^^c^	[12]^b^^,^^c^

a Calculated propagated errors.

b Quick test on 1 sample of each l-AEM (with sample available).

c Not measured at Surrey.

### The *ex situ* characterization of the Cl^−^-form l-AEMs (NREL protocols)^42^

The methods used at NREL differ from the laboratory at Surrey and are summarized in the next few sections.^42^ The values collected at NREL are compared to Surrey's values in Table 2 in the results section. To ensure the l-AEMs were in the Cl^−^-form after delivery, the l-AEMs were soaked in 100 cm^3^ of aqueous NaCl (1 mol dm^−3^) for 30 min, which was then repeated three more times with fresh solution. The l-AEM samples were then washed with 100 cm^3^ of deionized water for 30 min, which was again repeated three more times with fresh UPW. The Cl^−^-ion-exchanged l-AEMs were then dried for a minimum of 12 h in a laboratory convection oven exposed to air at 60 °C.

For the measurement of IEC, dry Cl^−^-exchanged l-AEM samples were weighed (dry weight of the sample must be >50 mg for adequate precision) and then each was soaked in 60 cm^3^ of aqueous NaNO_3_ (0.1 mol dm^−3^) solution for a minimum of 6 h. Each solution, still containing the AEM sample, was titrated with aqueous AgNO_3_ (0.1 mol dm^−3^) solution using a Mettler Toledo (MT) T90 auto-titrator fitted with a DM141-SC sensor. Measurements were performed twice per l-AEM and the IECs were calculated using eqn (2).

For the WU measurements, the Cl^−^-exchanged l-AEM samples were cut into squares (surface area = 5 cm^2^) using a die cutter and submerged in deionized water at room temperature for 24 h. The hydrated samples were patted dry using Kimwipe tissue paper and immediately weighed. Three wet thickness measurements were then immediately recorded from the centre of each l-AEM sample using a micrometer and averaged. The samples were then dried for 12 h minimum in a laboratory convection oven exposed to air at 60 °C before being weighed again, immediately upon removal from the oven. Three dry thickness measurements were then immediately recorded from the centre of each l-AEM sample using a micrometer and averaged. The WU, TPS, and *λ*(H_2_O) values were calculated using eqn (3)–(5), respectively. The length swelling (%) in the *x*-direction was also measured (the *x*-direction being the direction of largest swelling when starting with a dry perfectly square sample of l-AEM).

### The measurement of Cl^−^ conductivities of l-AEMs in water (Surrey protocols)^33^

The Cl^−^ conductivities of the l-AEMs in water were recorded using the protocol reported previously.^33^ In summary, each sample of Cl^−^-form l-AEM was mounted in a four-point BekkTech BT-112 cell (supplied by Alvatek, UK), which was then submerged in UPW at controlled increasing temperatures (minimum of 20 min at each temperature). The resistance of the sample was recorded as the low-frequency intercept of an electrochemical impedance spectrum (EIS) recorded at frequencies ranging 0.3 Hz to 100 kHz (EIS recorded on a Solartron 1260–1287 instrument combination, 10 mV a.c. amplitude, 0 V d.c. bias). The in-plane conductivities (*σ*/S cm^−1^) were calculated using eqn (6):6
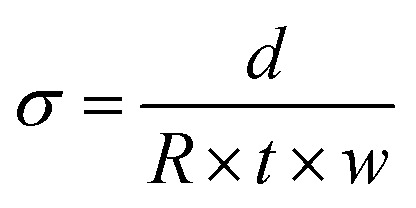
where *R* = resistance (Ω), *d* = distance between the inner Pt-sense wires (cm), *w* = width of the l-AEM sample (cm), and *t* = thickness of the l-AEM sample under the same conditions (cm).

### The measurement of Cl^−^ conductivities of l-AEMs at RH = 95% (NREL protocols)^42^

Each l-AEM sample (5 mm wide × 30 mm long) was placed in a four-point in-plane conductivity cell with four parallel Pt electrodes (alternating current is applied to the outer electrodes and the a.c. voltage response measured between the inner potential sense wires). Ionic resistance was measured from EIS recorded using a Solartron 1470E 8-channel potentiostat combined with a 1400A frequency response analyzer (frequency range of 10–105 Hz, 10 mV a.c. amplitude, 0 V d.c. bias). The conductivity cells were mounted in an environmental chamber so that resistances could be recorded at increasing temperatures (30–80 °C) at relative humidity RH = 95% (60 min at each temperature), or at different RHs (40–95%) at 80 °C (75 min at each humidity). The in-plane conductivities (*σ*/mS cm^−1^) were calculated using eqn (6).

### The measurement of alkali stabilities of the l-AEMs in aqueous KOH (1 mol dm^−3^) at 80 °C (NREL screening protocol)^42^

The AEM samples used for IEC determinations were dried overnight at 60 °C in a convection oven exposed to ambient air to obtain a total AEM dry mass between 100–150 mg. The samples were individually placed in 20 cm^3^ Teflon-lined Parr reactors, after which 10 cm^3^ of a freshly made solution of aqueous KOH (1 mol dm^−3^) was added, with special attention to ensuring the entire AEM sample was covered by the solution. The reactors were tightly closed and placed within an oven at 80 °C for 1000 h. After this time, the reactors were removed and quenched in cool water. The samples were removed and washed with UPW. The AEM samples were exchanged back to the Cl^−^-forms (according to the previously described protocol). The post-degradation IECs and Cl^−^ conductivities were then measured (according to the NREL protocols described above). The conductivity and IEC losses on degradation were calculated using eqn (7):7
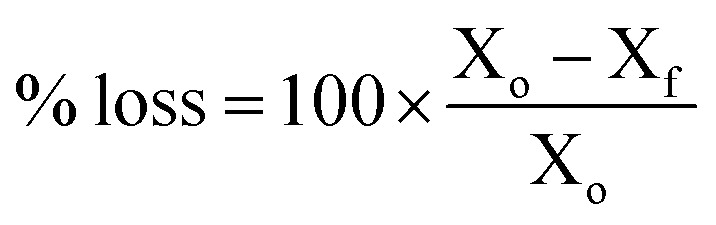
where X_o_ = the property (IEC or Cl^−^ conductivity) before degradation and X_f_ = the property after alkali ageing (degradation). Optical photographs of the samples before and after degradation were also recorded to give a visual assessment of mechanical integrity.

### The measurement of alkali stabilities of the OH^−^-form l-AEMs at RH = 95% at 80 °C (MPI-FKF protocols)

The l-AEMs were converted to the OH^−^ form by immersing the Cl^−^-forms in excess aqueous NaOH (1 mol dm^−3^) at ambient temperature in a CO_2_-free environment. The solution was replaced by a fresh solution 4 times during a total ion exchange period of 3 d. Residual NaOH in the membrane was washed out in UPW which was also replaced 4 times over 3 d.

Stability tests at high humidification (RH = 95%) were done in a vertical arrangement of two stainless steel vessels, each about 1 dm^3^ in volume connected by a short pipe (5 cm long). The bottom vessel, half filled with water to serve as a humidifier, was kept at a temperature of 78.5 °C. The upper vessel was controlled at a temperature 80 °C and contained the OH^−^-form l-AEM under test, which was placed in a small glass beaker to guarantee identical degradation conditions. CO_2_-free N_2_ was passed through the system from the bottom (humidifier) to the top (sample vessel) at a rate of 40 cm^3^ min^−1^, leaving the device through a gas bubbler. The 1000 h test duration required the refilling of the humidifier during operation through a vertical pipe. To avoid cold spots, the two vessels were packed in thermally conducting copper-foil and the transition pipe was heated to 85 °C. The IEC of each type of l-AEM was determined as described above (three independent titrations before and after the test) using the masses of the dry Cl^−^-forms in eqn (2). The IECs of the OH^−^-forms (unstable in the dry state) can be calculated using 18.4 g mol^−1^ as the mass difference between the two counter anions.

Additionally, there was enough l-AEM-TMA sample to conduct a long-term room-temperature storage test. This sample (ion-exchanged to the OH^−^-form in June 2018) was stored in the OH^−^-form in a CO_2_-free glovebox in CO_2_-purged UPW until February 2020 (21 months). The post-storage IECs were then compared with the pre-storage IECs.

### The measurement of alkali stabilities of the OH^−^-form l-AEMs at RH = 50% using a thermogravimetric analysis method (MPI-FKF)^38^

Details of this method were published previously,^38^ using a balance with magnetic coupling as previously described.^43^ Samples of OH^−^-form AEM (100–200 mg) were placed in a quartz crucible within a CO_2_-free glovebox and then transferred to the balance using a gas tight glass container. N_2_ gas was passed through a CO_2_ absorber (sodalime with indicator, Merck no. 1.06733.0501) before entering the humidification system of the balance. Stability tests were carried out at controlled temperatures and at RH = 50%. In all experiments, the decomposition intervals were 20 h interrupted by 5 h intervals of higher RH = 65% to allow the transient IEC to be calculated.^38^ After TGA testing (*ca.* 200 h), samples were exchanged back into the Cl^−^-form (submersion in excess aqueous NaCl (1 mol dm^−3^) for 5 d at 40 °C) before determining the residual IEC as described previously.^38^ Testing was also conducted at RH = 10%.^38^

## Results and discussion

### Raman spectroscopic confirmation of l-AEM synthesis

**Fig. 1:**
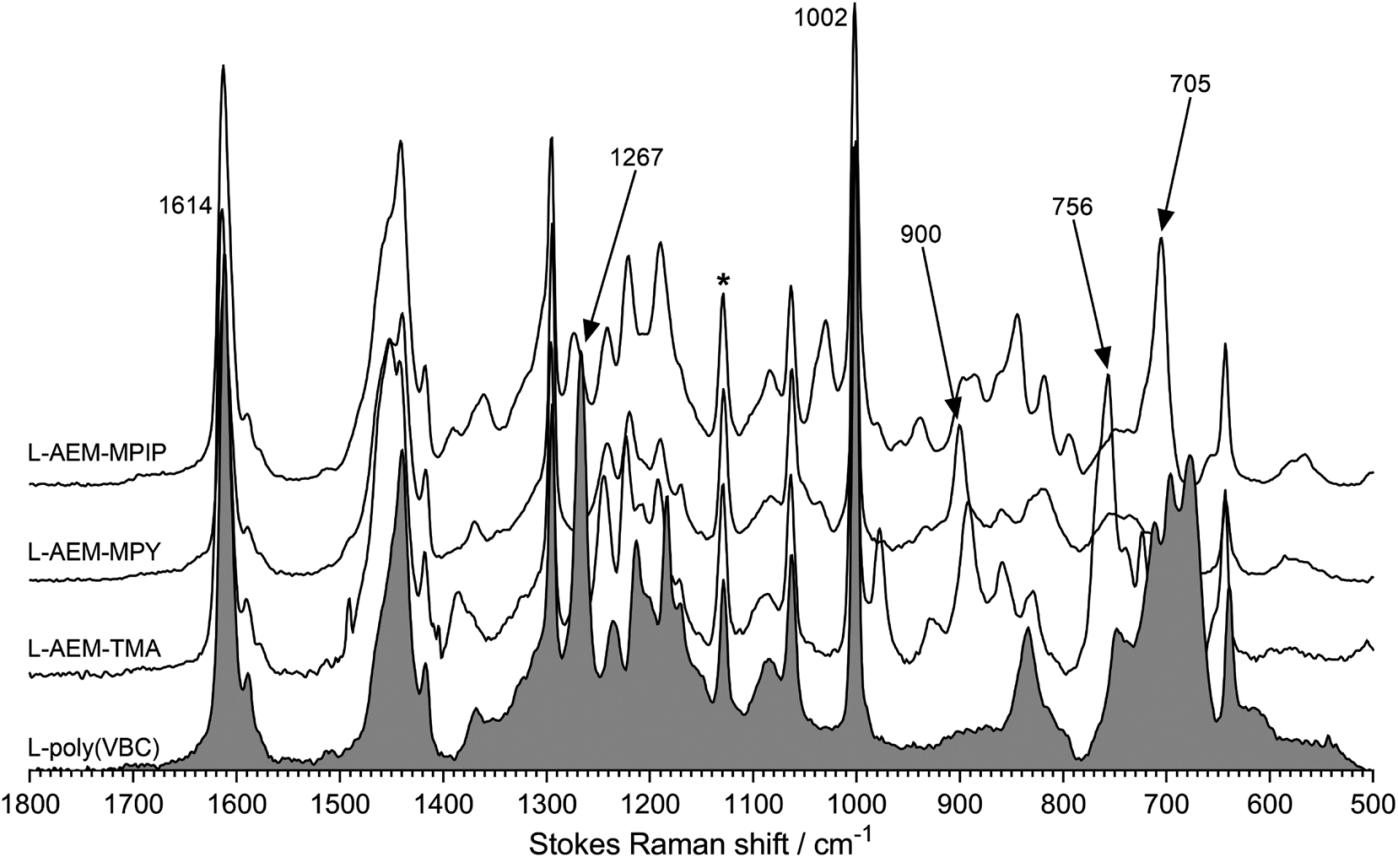
The Raman spectra of the l-AEMs and the pre-aminated poly(VBC)-grafted membrane (shaded spectrum). * indicates the LDPE-derived peak used to normalize the intensities of the spectra to aid visual comparison. Spectra recorded with a laser *λ* = 532 nm.

Characterization data has been published on these classes of RG-AEMs.^31,34^ Raman spectra were recorded to check for successful l-AEM synthesis (Fig. 1). The spectrum of the pre-aminated l-poly(VBC) membrane consisted of the expected superposition of the peaks due to the LDPE precursor film and the peaks due to the grafted poly(VBC) chains; the latter includes a characteristic peak at 1269 cm^−1^ (confirms the presence of the –CH_2_Cl groups on the grafted poly(VBC) chains) and the expected aromatic peaks at 1614 and 1002 cm^−1^. For all l-AEMs, the 1267 cm^−1^ peak disappeared after amination, whilst quaternary ammonium peaks appeared that are characteristic for benzyltrimethylammonium, benzyl-*N*-methylpyrrolidinium, and benzyl-*N*-methylpiperidnium groups (*e.g.* 756 cm^−1^, 900 cm^−1^, and 705 cm^−1^, respectively), as previously reported.^30,31^

To check for grafting homogeneity, Raman spectra were recorded on five random spots on the surfaces of the pre-aminated l-poly(VBC) membranes. The ratio between the integrated area of the 1614 cm^−1^ peak (due to the aromatic ring breathing in the poly(VBC) grafted chains) normalised to the area of 1130 cm^−1^ peak (C–C stretch from the LDPE chains) was calculated from each spectrum to be 7.90 ± 0.23 (sample standard deviation, *n* = 5). As the relative standard deviation (RSD) was <3%, grafting homogeneity was deemed to be very good.^44^

### IECs of the l-AEMs as synthesized (in Cl^−^-forms)

The IECs of the l-AEMs are presented in Table 2. As expected, the IECs are lower for the MPY- and MPIP-based head-groups compared to the TMA-based materials (due to the heterocyclic groups having higher molar masses compared to the benchmark TMA-based chemistry). The IECs of the l-AEMs (measured in the Cl^−^ anion forms) ranged 2.35–2.68 mmol g^−1^ when measured at Surrey, 2.26–2.48 mmol g^−1^ when measured at NREL, and 2.43–2.72 mmol g^−1^ when measured at MPI-FKF. Differences of <10% are reasonable for independent tests for IEC using different apparatus. The IEC of l-AEM-TMA is in reasonable agreement with the IEC (Cl^−^) data that was previously reported with a different batch of TMA-based RG-AEM made by a different researcher using the same 25 μm LDPE precursor and a similar synthesis protocol (2.87 ± 0.05 mmol g^−1^).^33^

### Water contents of the l-AEMs (in Cl^−^ forms)

Table 2 summarises select key properties of the l-AEMs. The l-AEM-MPY and l-AEM-MPIP did not physically swell more than the l-AEM-TMA (within the large errors present in these simple measurements). However, the water contents and thicknesses of the fully hydrated l-AEMs (in water) follow the trend l-AEM-MPIP > l-AEM-MPY > l-AEM-TMA, despite the lower IECs of l-AEM-MPIP and l-AEM-MPY. This trend was observed in the data collected at both Surrey and NREL and matches the trend observed with previously reported ETFE-based RG-AEMs containing these head-group chemistries.^30^ This is promising in relation to the stabilities of the l-AEMs in high pH conditions; current thinking is that maintaining higher hydration levels in AEMs helps reduce the nucleophilic aggressiveness of the OH^−^ anions.^45^

**Fig. 2:**
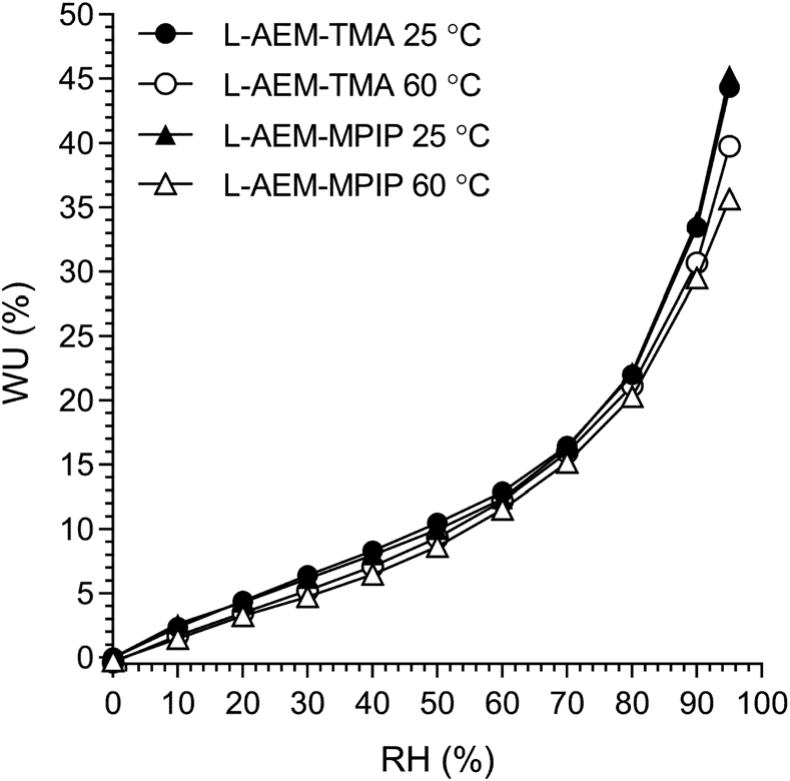
The humidified gravimetric water uptakes (WU) at 25 and 60 °C for LDPE-AEM-TMA and LDPE-AEM-MPIP in the Cl^−^-forms. WU values are the averages from the hydration and dehydration cycles during the dynamic vapor sorption (DVS) experiments. Data collected at NREL.

However, AEMs will not be submerged in H_2_O in operating AEMFCs. Hence, the lowest and highest water content AEMs (l-AEM-TMA and l-AEM-MPIP, respectively) were evaluated for dynamic vapor sorption (DVS) at both 25 and 60 °C (we did not have enough of this exact batch of l-AEM-MPY to conduct a DVS test). It is clear from Fig. 2, that the WU values recorded at all RHs and both temperatures were highly similar for both the l-AEMs tested. Even at RH = 95%, the WU values were significantly lower (<50%) than those recorded in water (Table 2, >100%). This may have important implications related to the relative differences in the conductivities and alkali stabilities of the different chemistry l-AEMs (see discussions later).

### Cl^−^ conductivities of the l-AEMs

**Fig. 3:**
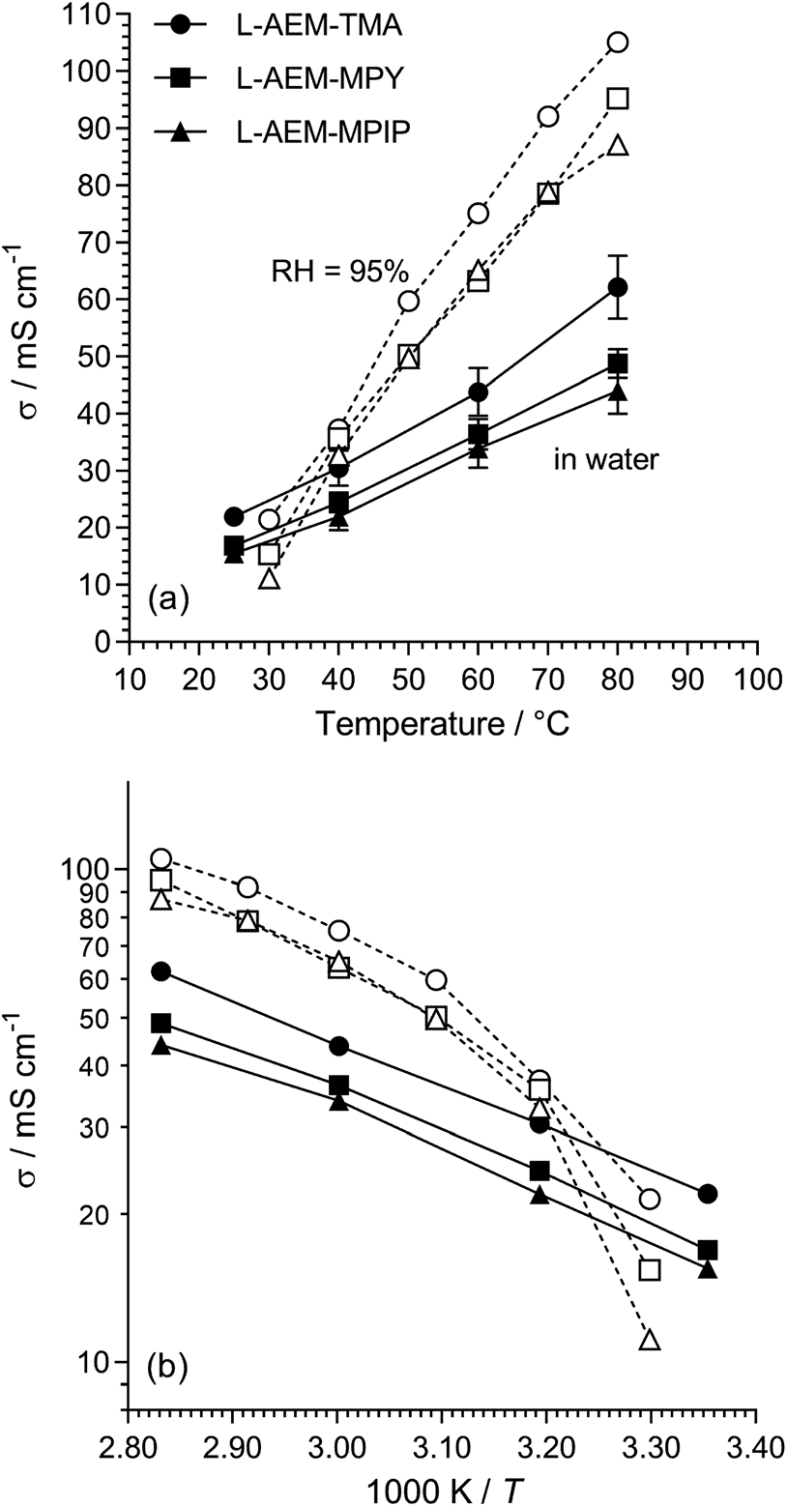
(a) The Cl^−^ conductivities of the l-AEMs in water (black symbols, measured at Surrey using the 4-probe (in-plane) method, 20 min at each temperature before measurement taken) and at RH = 95% (open symbols, measured at NREL using the 4-probe (in-plane) method in an environmental chamber, 60 min at each temperature before measurement taken). The error bars are sample standard deviations from repeat measurements on *n* = 3 different samples of each AEM. (b) The associated log plots (error bars omitted for clarity).

The Cl^−^ conductivities of the l-AEMs were recorded in water (at Surrey) and in RH = 95% humidities (at NREL). The data obtained are presented in Fig. 3. Surprisingly, the conductivities at temperatures >40 °C were higher in a RH = 95% atmosphere than when the l-AEMs were submerged in water. This suggests that the excessive water uptakes (108–187 wt%) when the l-AEMs are submerged in water is severely diluting the concentration of charge carriers, leading to lower conductivities. The data shows that l-AEM-TMA has the highest conductivities under all conditions tested.

For the water measurements, the activation energies (*E*_a_/kJ mol^−1^) were 16.5, 16.9 and 16.9 for l-AEM-TMA, l-AEM-MPY, and l-AEM-MPIP respectively (assuming simple Arrhenius behavior of ln(*σ*) *vs.* 1/*T*). This compares to *E*_a_ values ranging 16–20 kJ mol^−1^ for the previously reported ETFE-based TMA-AEMs of various IECs in water.^32^ The temperature dependence on the conductivity of the l-AEMs at RH = 95% suggests possible changes in hydration of the AEMs on heating in the humidified atmosphere (note this curvature does not fit VTF behaviour).

**Fig. 4:**
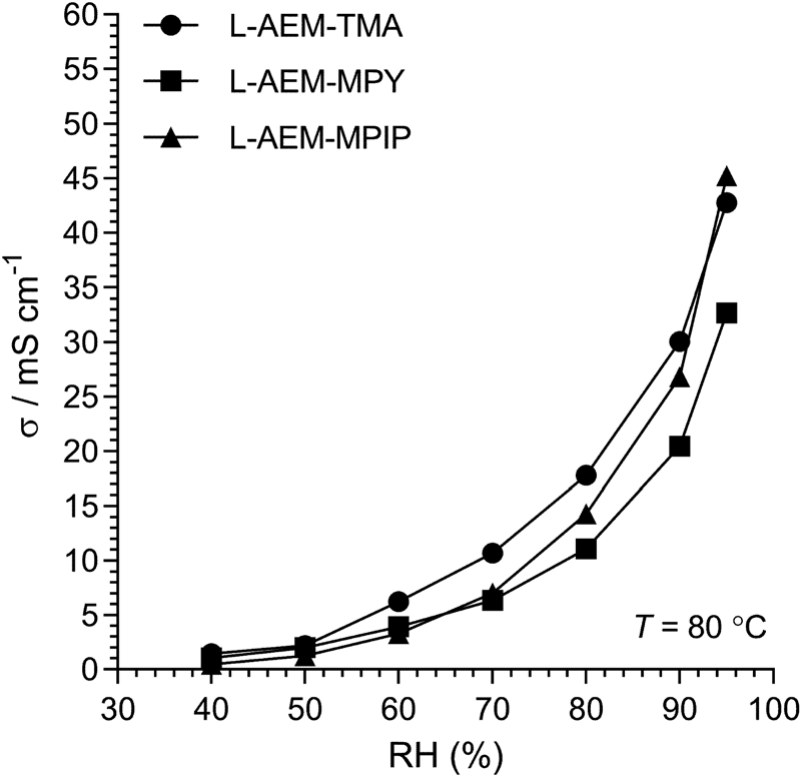
The Cl^−^ conductivities of the l-AEMs at 80 °C in atmospheres exposed to increasing RH (starting at RH = 40% and maintaining the samples at each RH for 75 min). Data collected at NREL.

The Cl^−^ conductivities of the l-AEMs were also determined at different RHs at 80 °C (Fig. 4). With increasing RH, the conductivities increase accordingly. However, the conductivities in the RH sweeps do not reach the conductivities obtained with the temperature sweeps (data in Fig. 3) under the same conditions. The highest conductivity during the RH sweep only reached 45 mS cm^−1^ at 95% RH and 80 °C (*cf.* 105 mS cm^−1^ at 95% RH and 80 °C in Fig. 3). This indicates a high level of non-equilibrium behavior, such that the l-AEMs may require long periods of time to become fully hydrated after being initially dehydrated to 40% RH.

### The alkali stabilities of the l-AEMs under different ex situ test conditions

#### Alkali stability testing in aqueous KOH at 80 °C for 1000 h

**Fig. 5:**
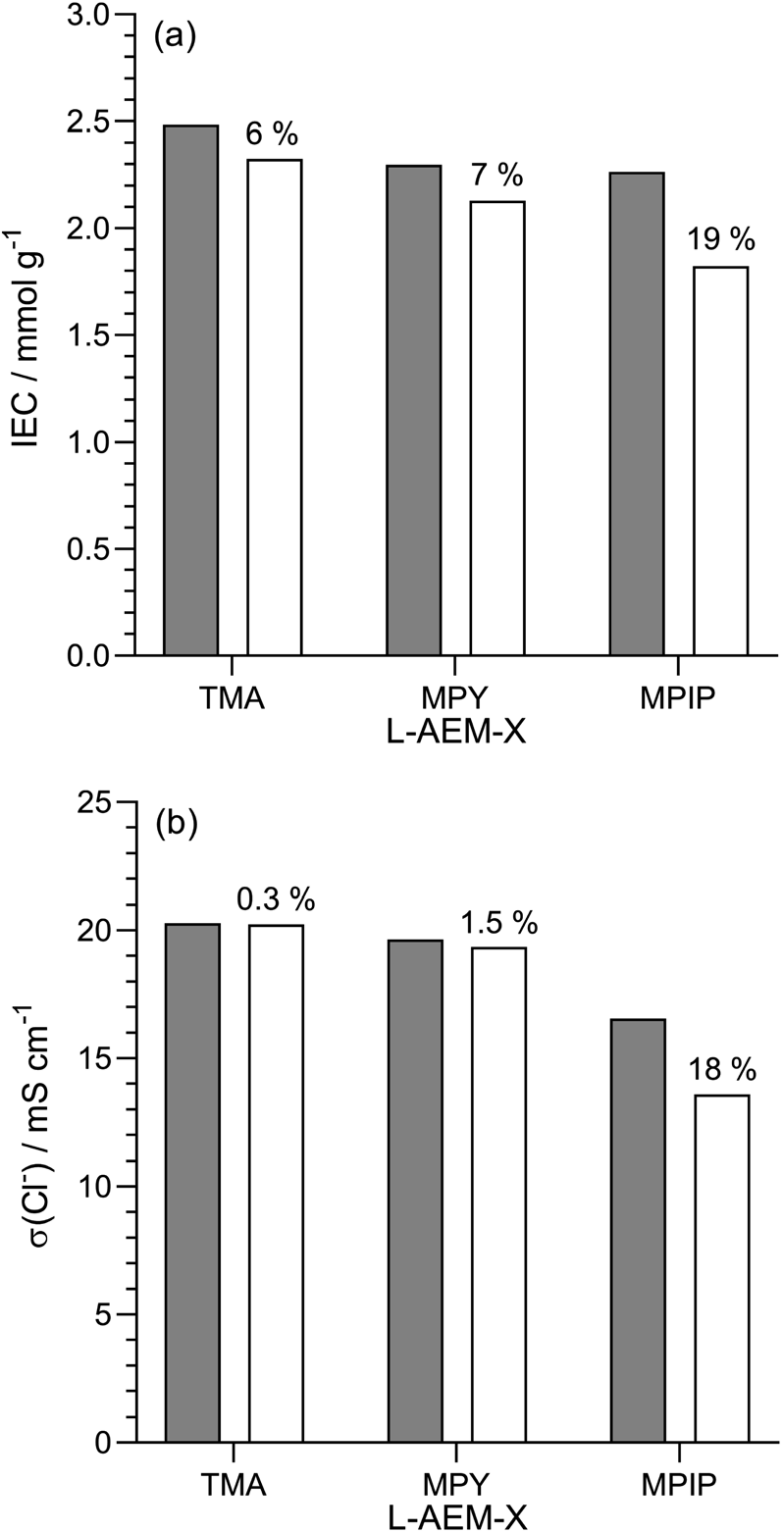
(a) IECs and (b) Cl^−^ conductivity (in water, room temperature) of the l-AEMs before (grey) and after (white) immersion in aqueous KOH (1 mol dm^−3^) at 80 °C for 1000 h. The values given above the white bars are the percentage loss of each property (calculated using eqn (7)). Data collected at NREL.

**Fig. 6:**
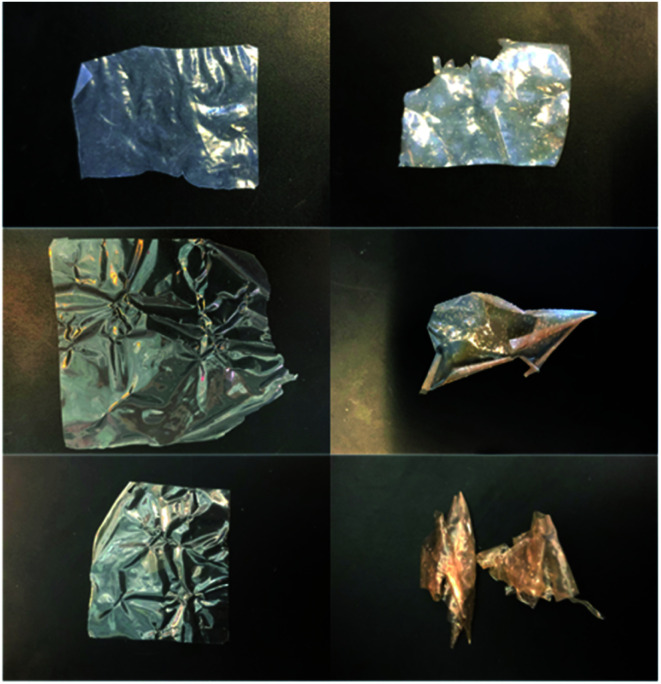
Photographs of the l-AEM samples before (left) and after (right) immersion in aqueous KOH (1 mol dm^−3^) at 80 °C for 1000 h: (top row) l-AEM-TMA, (middle row) l-AEM-MPY, and (bottom row) l-AEM-MPIP.

As highlighted in a recent opinion piece,^45^ most publications that study alkali stability involve measuring the changes in AEM properties when they are immersed in aqueous Na/KOH solutions of various concentrations and temperatures for various periods of time. This is clearly not representative of the *in situ* conditions in AEMFCs but is a useful tool for quickly screening out AEMs that have high instabilities towards alkali. Hence, we initially used NREL's standard test protocol and looked at the stability of the l-AEMs in aqueous KOH (1 mol dm^−3^) at 80 °C for 1000 h (Fig. 5). Losses of IEC were generally larger than the resulting losses in conductivity (the latter is a complex function of ion-solvation, affecting ion mobilities, and AEM swelling, affecting ion concentration and the distances for the ions to travel). l-AEM-MPIP was the least stable AEM in these tests. For the previously reported ETFE-based RG-AEMs,^30^ the MPY- and MPIP-based headgroups degraded less (IEC) than the TMA-based headgroups; however, these ETFE-based AEMS had much lower IECs and much larger differences between the IECs of the TMA- and MPY-/MPIP-headgroups, so a direct comparison with the l-AEMs is difficult. Post -degradation, l-AEM-TMA also retained more of its physical form (Fig. 6). As discussed previously, small molecule cycloaliphatic heterocyclic quaternary ammonium cations show relatively high stability towards OH^−^ attack when not attached to benzyl groups;^46^ benzylic attachment increases the rate of nucleophilic attack by at least an order of magnitude, which is in accordance with the present observation.

#### Alkali stability testing of the OH^−^-form l-AEMs in CO_2_-free RH = 95% at 80 °C

**Fig. 7:**
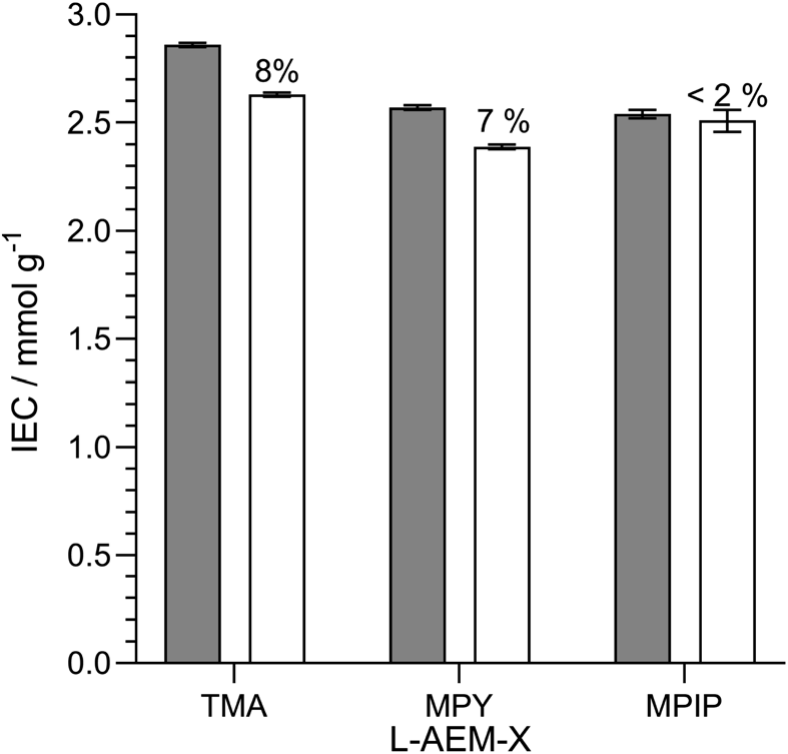
The IECs of the l-AEMs in the OH^−^-forms before (grey) and after (white) 1000 h of thermal treatment at 80 °C in a CO_2_-free RH = 95% atmosphere. The values given above the white bars are the percentage loss of IEC (calculated using eqn (7)). Data collected at the MPI-FKF.

AEMs are not immersed in aqueous alkali when being operated in AEMFCs. Hence, the next test we did was to look at changes in IEC when the l-AEMs in OH^−^-form were heated to 80 °C in a CO_2_-free (to avoid carbonation), high hydration (RH = 95%) atmosphere for 1000 h (Fig. 7). Here, l-AEM-MPIP appeared to proportionally lose the least amount of IEC (in contrast to the data in Fig. 5). This shows that head-group stability rankings can be highly dependent on the alkali degradation test conditions used.

#### Alkali stability testing of the OH^−^-form l-AEMs using a thermogravimetric analysis (TGA) method^38^

**Fig. 8:**
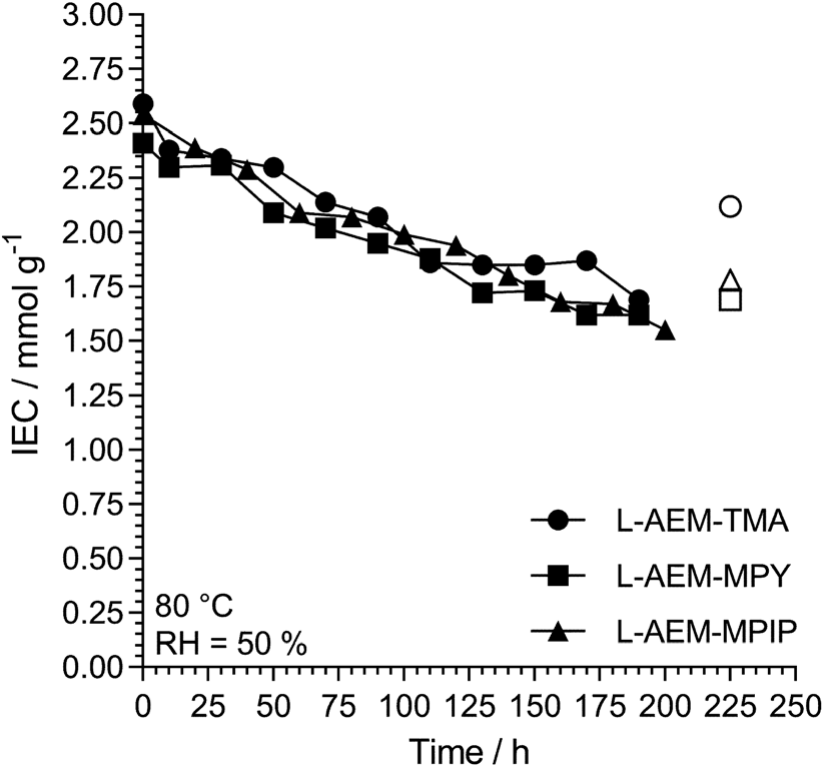
The alkali stability of the l-AEMs in the OH^−^-form at 80 °C in a CO_2_-free RH = 50% atmosphere measured using the TGA method described previously.^38^ The open symbols give the directly (titration) measured IECs recorded on the samples after the TGA experiments. Data collected at the MPI-FKF.

Maintaining high hydration can be challenging and can limit the operating range of the device. Hence, we also evaluated the alkali stabilities of the OH^−^-form l-AEMs at 80 °C in CO_2_-free RH = 50% environments using a previously reported TGA method (Fig. 8).^38^ The degradations for all l-AEMs were highly similar under these lower hydration conditions (although it appears that the TGA-derived data is slightly over-estimating the loss of IEC in the l-AEMs, compared to post-test titration-based IEC determinations).^38^

**Fig. 9:**
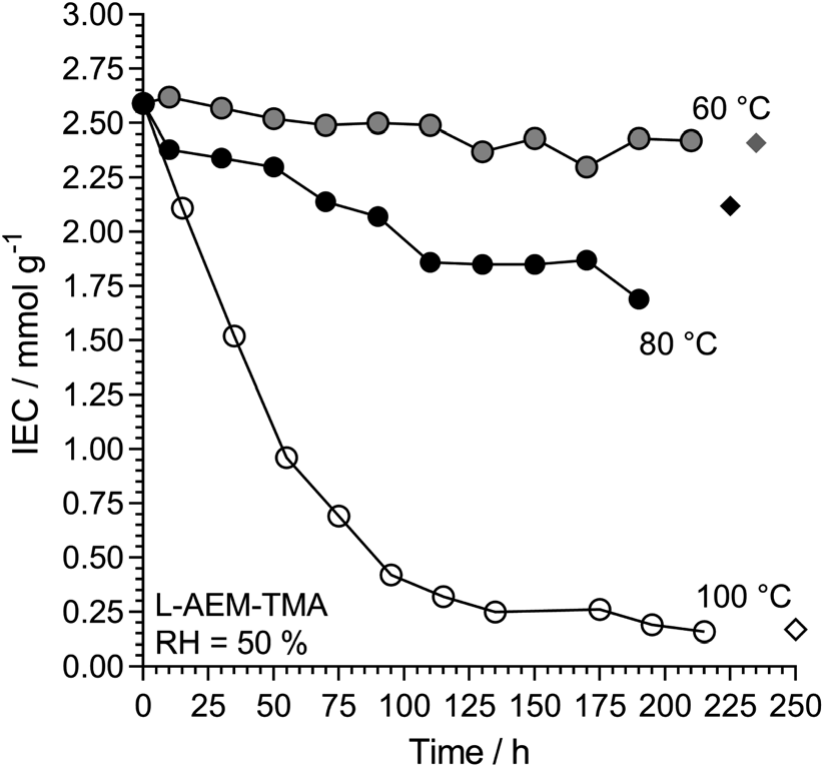
The alkali stability of l-AEM-TMA in the OH^−^-forms at various temperatures in a CO_2_-free RH = 50% atmosphere measured using the TGA method described previously (circles).^38^ The corresponding diamonds give the directly (titration) measured IECs recorded on the samples after the TGA experiments. Data collected at the MPI-FKF.

**Fig. 10:**
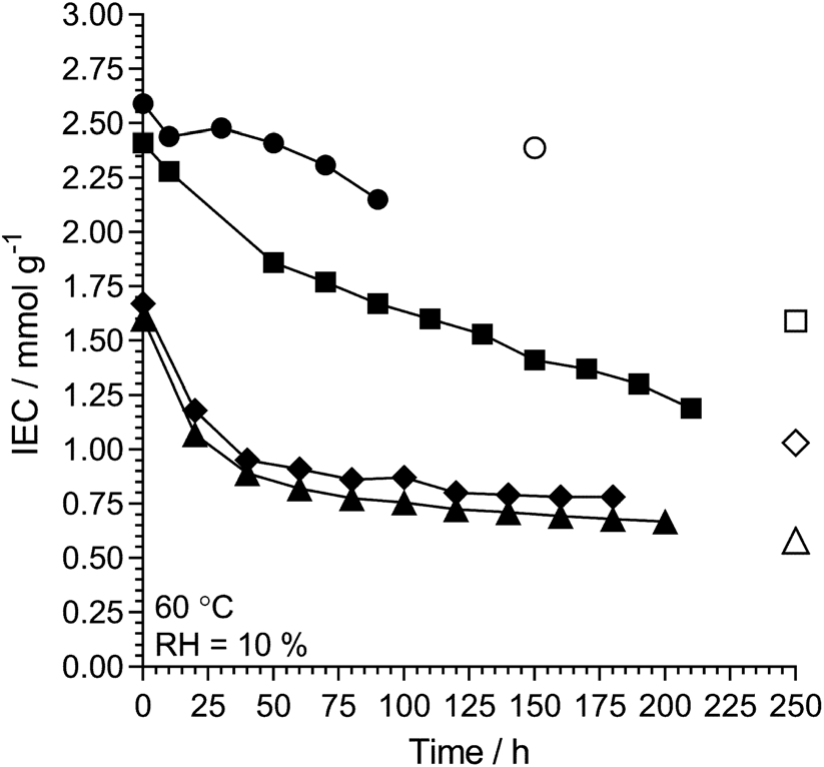
The alkali stability of l-AEM-TMA (black circles) and l-AEM-MPY (black squares) in the OH^−^-forms at 60 °C in a CO_2_-free RH = 10% atmosphere measured using the TGA method described previously.^38^ This data is compared to prior published polyphenylene- (black diamonds)^62^ and PPO-based (black triangles)^38^ AEMs tested under the same conditions. The corresponding open symbols give the directly (titration) measured IECs recorded on the samples after the TGA experiments. Data collected at the MPI-FKF.

We then investigated l-AEM-TMA (the highest conductivity type) in CO_2_-free RH = 50% environments at different temperatures (Fig. 9). Over the years, anecdotal evidence has been building in our laboratories that TMA-based RG-AEMs possess reasonable alkali stabilities under many test conditions at 60 °C, and these results support this. Degradation is more rapid at RH = 50% when the temperature is raised to 80 °C, while operating these AEMs at 100 °C at reduced RHs is not advised! As well as AEM water content and water diffusivity being critical for high OH^−^ conductivities and high AEMFC performances,^47–53^ hydration is also key to enhancing the alkali stabilities of AEMs.^45,54–60^ Note, a very recent result shows that LDPE-TMA-based RG-AEMs can be successfully employed in AEMFCs at 125 °C for reasonable periods of time when suitably hydrated.^61^

l-AEM-TMA and l-AEM-MPY were also tested at 60 °C in a RH = 10% TGA test (Fig. 10, note – there was not enough sample of the specific l-AEM-MPIP batch to do this RH = 10% experiment) alongside data for two different types of prior reported AEMs.^38,62^ Maintaining a residual IEC of >1 mmol g^−1^ under such severe dehydration conditions again shows that RG-AEMs are reasonably stable at 60 °C under a wide range of hydration conditions.

#### Final comparison of the changes in IEC of the l-AEMs on alkali degradation at 80 °C by the three different methods

**Fig. 11:**
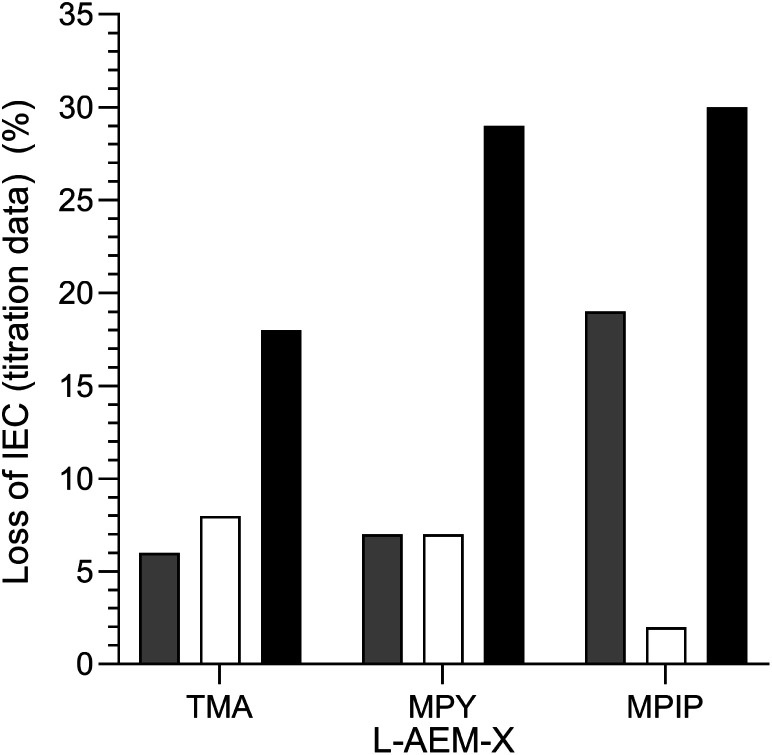
A summary of the *ex situ* 80 °C alkali degradation data obtained with the l-AEMs and the three different methods used in this study: (grey) the aqueous KOH method in Fig. 5; (white) the RH = 95% method in Fig. 7; and (black) the RH = 50% TGA method in Fig. 8. The IEC losses were calculated using eqn (7) using the titration-based IEC data collected both before and after degradation (under the three different conditions).

The alkali stabilities at 80 °C, extracted from the titration-derived IECs in Fig. 5 (submerged in aqueous KOH), Fig. 7 (OH^−^-forms in RH = 95% environment), and Fig. 8 (OH^−^-forms in RH = 50% environment), are compared in Fig. 11. The stabilities of the different head-groups differ by test conditions but, overall, there does not appear to be any overriding benefit (conductivity or chemical stability wise (IEC)) in replacing the TMA-type head-group with MPY- and MPIP-types for this class of LDPE-based RG-AEMs. It is important to note that the highest degradation rates are consistently observed for RG-AEMs in the OH^−^-forms under reduced RH conditions (TGA experiment), which are quasi-representative to the conditions in a running AEMFC, *e.g.* close to the cathodes at high current densities or during transient temperature excursions. Recall, the commonly used aqueous KOH stability tests (where OH^−^ anions are under highly hydrating conditions) can underestimate the AEM degradation in operating AEMFCs.^45,56,62^

### A comment on anion-exchange ionomer (AEI) degradation

Obviously, these *ex situ* determinations of alkali stabilities will need to be correlated to *in situ* stabilities in due course, but this is not straightforward: *in situ* AEMFC durabilities are a function not just of the AEM but also of the AEI, catalyst, and catalyst support in the electrodes, including the direct effect of each catalyst on AEI degradation (*e.g.* oxidation of phenyl groups in the AEI).^63,64^ In fact, the general thinking is that AEMFC performance degradation over even short timeframes is mostly affected by either degradation of the alkali ionomer in the cathode (where dehydration is severe even at moderate current densities) or flooding events in the electrodes.^3,47^ Continued research is required in order to satisfactorily decouple the AEM, AEI and catalyst degradations and the flooding events in *operando* AEMFCs.

### Long-term storage of the OH^−^-form l-AEM-TMA in water

For long-term storage, it is recommended practice to store the RG-AEMs in water in the Cl^−^-forms (not the OH^−^-forms). The MPI-FKF team stored a sample of OH^−^-form l-AEM-TMA in CO_2_-free UPW in a CO_2_-free glovebox environment for 21 months at room temperature. The initial IEC was 2.82 mmol g^−1^ (calculated with the mass of the OH^−^ form), which dropped to 2.64 mmol g^−1^ after this period of long-term storage. This represents a 6% loss of IEC (*ca.* a loss of 0.009 mmol g^−1^ month^−1^, assuming a linear loss of IEC with time), whereas Surrey has observed no loss in IEC over many years when different RG-AEMs are stored in water in the Cl^−^-forms.

The colouration of the l-AEM-TMA after 21 months storage in the OH^−^-form was not too extreme, which allowed post-mortem Raman analysis (the usual post-degradation darkening of RG-AEMs prevents the collection of Raman spectra with good enough quality for quantitative analysis due to photoluminescence effects). Hence, the Raman spectra of the post-storage sample (*n* = 5 spectra taken on random spots on both sides, converted back to the Cl^−^-form, *λ* = 532 nm) were compared to the spectra of the as-synthesised sample (*n* = 6 spectra taken on random spots on both sides, Cl^−^-form, *λ* = 532 nm). The areas of the benzyltrimethylammonium-derived peaks at 756 cm^−1^ normalised to the areas of the LDPE-derived peaks at 1130 cm^−1^ gave the following ratios: 2.03 ± 0.15 (relative standard deviation = 8%) for the post-storage sample and 2.28 ± 0.25 (relative standard deviation = 11%) for the as-synthesised samples. This represents a degradation of *ca.* 5% (calculated using eqn (7)) in good agreement with the 6% loss of IEC on long-term storage.

## Conclusions

In summary, trimethylammonium-(TMA)-type low-density polyethylene-(LDPE)-based radiation-grafted anion-exchange membranes (l-AEM) generally exhibit higher conductivities and lower levels of alkali degradation under a variety of *ex situ* degradation conditions, compared to *N*-methylpyrrolidinium-(MPY)- and *N*-methylpiperidinium-(MPIP)-analogues, especially at lower hydration levels. Such RG-AEMs should be submerged in water in the Cl^−^-forms for any long-term storage (post-synthesis). l-AEMs can have very high conductivities even in the Cl^−^ forms when in highly humidified atmospheres.

## Authors contribution

Carly Reed made the l-AEMs during her final year undergraduate project at Surrey, under the daily supervision of Dr Rachida Bance-Soualhi, and conducted all of the Surrey-based characterisations. Dr Kelly Meek conducted all of the NREL-based characterisations under the supervision of Dr Bryan Pivovar. Prof Klaus-Dieter Kreuer supervised all of the MPI-FKF-based experiments, including data interpretation. Prof Varcoe co-ordinated the study and drafted the main bulk of this paper.

## Conflicts of interest

There are no conflicts to declare.

## Supplementary Material

## References

[cit1] Varcoe J. R., Atanassov P., Dekel D. R., Herring A. M., Hickner M. A., Kohl P. A., Kucernak A. R., Mustain W. E., Nijmeijer K., Scott K., Xu T., Zhuang L. (2014). Energy Environ. Sci..

[cit2] Gottesfeld S., Dekel D. R., Page M., Bae C., Yan Y., Zelenay P., Kim Y. S. (2018). J. Power Sources.

[cit3] Peng X., Kulkarni D., Huang Y., Omasta T. J., Ng B., Zheng Y., Wang L., LaManna J. M., Hussey D. S., Varcoe J. R., Zenyuk I. V., Mustain W. E. (2020). Nat. Commun..

[cit4] Zhegur-Khais A., Kubannek F., Krewer U., Dekel D. R. (2020). J. Membr. Sci..

[cit5] Tatus-Portnoy A., Kitayev A., Vineesh T. V., Tal-Gutelmacher E., Page M., Zitoun D. (2020). Chem. Commun..

[cit6] Thompson S. T., Peterson D., Ho D., Papageorgopoulos D. (2020). J. Electrochem. Soc..

[cit7] Truong V. M., Tolchard J. R., Svendby J., Manikandan M., Miller H. A., Sunde S., Yang H., Dekel D. R., Barnett A. O. (2020). Energies.

[cit8] Firouzjaie H. A., Mustain W. E. (2020). ACS Catal..

[cit9] Dekel D. R. (2018). J. Power Sources.

[cit10] Wang J., Zhao Y., Setzler B. P., Rojas-Carbonell S., Yehuda C. B., Amel A., Page M., Wang L., Hu K., Shi L., Gottesfeld S., Xu B., Yan Y. (2019). Nat. Energy.

[cit11] Wang L., Peng X., Mustain W. E., Varcoe J. R. (2019). Energy Environ. Sci..

[cit12] Huang G., Mandal M., Peng X., Yang-Neyerlin A. C., Pivovar B. S., Mustain W. E., Kohl P. A. (2019). J. Electrochem. Soc..

[cit13] Fan J., Willdorf-Cohen S., Schibli E. M., Paula Z., Li W., Skalski T. J. G., Tersakian Sergeenko A., Hohenadel A., Frisken B. J., Magliocca E., Mustain W. E., Diesendruck C. E., Dekel D. R., Holdcroft S. (2019). Nat. Commun..

[cit14] Abbasi R., Setzler B. P., Lin S., Wang J., Zhao Y., Xu H., Pivovar B., Tian B., Chen X., Wu G., Yan Y. (2019). Adv. Mater..

[cit15] Wang Z., Sankarasubramanian S., Ramani V. (2018). Curr. Opin. Electrochem..

[cit16] Yin Z., Peng H., Wei X., Zhou H., Gong J., Huai M., Xiao L., Wang G., Lu J., Zhuang L. (2019). Energy Environ. Sci..

[cit17] Zhu Y., Ding L., Liang X., Shehzad M. A., Wang L., Ge X., He Y., Wu L., Varcoe J. R., Xu T. (2018). Energy Environ. Sci..

[cit18] Peng H., Li Q., Hu M., Xiao L., Lu J., Zhuang L. (2018). J. Power Sources.

[cit19] Ayers K., Danilovic N., Ouimet R., Carmo M., Pivovar B., Bornstein M. (2019). Annu. Rev. Chem. Biomol. Eng..

[cit20] Espiritu R., Golding B. T., Scott K., Mamlouk M. (2017). J. Mater. Chem. A.

[cit21] Espiritu R., Tan J. L., Lim L. H., Arco S. (2020). J. Phys. Org. Chem..

[cit22] Mamlouk M., Horsfall J. A., Williams C., Scott K. (2012). Int. J. Hydrogen Energy.

[cit23] Danks T. N., Slade R. C. T., Varcoe J. R. (2002). J. Mater. Chem..

[cit24] Zhao Y., Yoshimura K., Takamatsu H., Hiroki A., Kishiyama Y., Shishitani H., Yamaguchi S., Tanaka H., Koizumi S., Radulescu A., Appavou M.-S., Maekawa Y. (2019). J. Electrochem. Soc..

[cit25] Sherazi T. A., Zahoor S., Raza R., Shaikh A. J., Naqvi S. A. R., Abbas G., Khan Y., Li S. (2015). Int. J. Hydrogen Energy.

[cit26] Deavin O. I., Murphy S., Ong A. L., Poynton S. D., Zeng R., Herman H., Varcoe J. R. (2012). Energy Environ. Sci..

[cit27] Park J. H., Han M. J., Song D. S., Jho J. Y. (2014). ACS Appl. Mater. Interfaces.

[cit28] Fang J., Yang Y., Lu X., Ye M., Li W., Zhang Y. (2012). Int. J. Hydrogen Energy.

[cit29] Svarfar B. L., Ekman K. B., Sundell M. J., Näsman J. H. (1996). Polym. Adv. Technol..

[cit30] Ponce-Gonzalez J., Whelligan D. K., Wang L., Bance-Soualhi R., Wang Y., Peng Y., Peng H., Apperley D. C., Sarode H. N., Pandey T. P., Divekar A. G., Seifert S., Herring A. M., Zhuang L., Varcoe J. R. (2016). Energy Environ. Sci..

[cit31] Gonçalves Biancolli A. L., Herranz D., Wang L., Stehlíková G., Bance-Soualhi R., Ponce-González J., Ocón P., Ticianelli E. A., Whelligan D. K., Varcoe J. R., Santiago E. I. (2018). J. Mater. Chem. A.

[cit32] Wang L., Maggliocca E., Cunningham E. L., Mustain W. E., Poynton S. D., Escudero-Cid R., Nasef M. M., Ponce-González J., Bance-Souahli R., Slade R. C. T., Whelligan D. K., Varcoe J. R. (2017). Green Chem..

[cit33] Wang L., Brink J. J., Liu Y., Herring A. M., Ponce-Gonzalez J., Whelligan D. K., Varcoe J. R. (2017). Energy Environ. Sci..

[cit34] Wang L., Bellini M., Miller H. A., Varcoe J. R. (2018). J. Mater. Chem. A.

[cit35] Peng X., Omasta T. J., Magliocca E., Wang L., Varcoe J. R., Mustain W. E. (2018). Angew. Chem., Int. Ed..

[cit36] Page O. M. M., Poynton S. D., Murphy S., Ong A. L., Hillman D. M., Hancock C. A., Hale M. G., Apperley D. C., Varcoe J. R. (2013). RSC Adv..

[cit37] Bharath V. J., Jervis R., Millichamp J., Neville T. P., Mason T., Tjaden B., Shearing P. R., Brown R. J. C., Manos G., Brett D. J. L. (2017). Int. J. Hydrogen Energy.

[cit38] Kreuer K. D., Jannasch P. (2018). J. Power Sources.

[cit39] Saleh A. S., Ibrahim A. G., Elsharma E. M., Metwally E., Siyam T. (2018). Radiat. Phys. Chem..

[cit40] Nasef M. M. (2014). Chem. Rev..

[cit41] Gubler L. (2014). Adv. Energy Mater..

[cit42] Meek K. M., Antunes C. M., Strasser D. J., Owczarczyk Z. R., Yang-Neyerlin A. C., Pivovar B. S. (2019). ECS Trans..

[cit43] Kreuer K. D. (2013). Solid State Ionics.

[cit44] Lee W. H., Crean C., Varcoe J. R., Bance-Soualhi R. (2017). RSC Adv..

[cit45] Diesendruck C. E., Dekel D. R. (2018). Curr. Opin. Electrochem..

[cit46] Marino M. G., Kreuer K. D. (2015). ChemSusChem.

[cit47] Yassin K., Rasin I. G., Brandon S., Dekel D. R. (2020). J. Membr. Sci..

[cit48] Schwämmlein J. N., Pham N. L. T., Mittermeier T., Egawa M., Bonorand L., Gasteiger H. A. (2020). J. Electrochem. Soc..

[cit49] Zadok I., Dekel D. R., Srebnik S. (2019). J. Phys. Chem. C.

[cit50] Mandal M., Huang G., Hassan N. U., Peng X., Gu T., Brooks-Starks A. H., Bahar B., Mustain W. E., Kohl P. A. (2020). J. Electrochem. Soc..

[cit51] Chen Z. (2020). Nat. Energy.

[cit52] Zelovich T., Vogt-Maranto L., Hickner M. A., Paddison S. J., Bae C., Dekel D. R., Tuckerman M. E. (2019). Chem. Mater..

[cit53] Zheng Y., Ash U., Pandey R. P., Ozioko A. G., Ponce-González J., Handl M., Weissbach T., Varcoe J. R., Holdcroft S., Liberatore M. W., Hiesgen R., Dekel D. R. (2018). Macromolecules.

[cit54] Dekel D. R., Amar M., Willdorf S., Kosa M., Dhara S., Diesendruck C. E. (2017). Chem. Mater..

[cit55] Pusara S., Srebnik S., Dekel D. R. (2018). J. Phys. Chem. C.

[cit56] Willdorf-Cohen S., Mondal A. N., Dekel D. R., Diesendruck C. E. (2018). J. Mater. Chem. A.

[cit57] Zhang W., Dong D., Bedrov D., van Duin A. C. T. (2019). J. Mater. Chem. A.

[cit58] Dekel D. R., Rasin I. G., Brandon S. (2019). J. Power Sources.

[cit59] Han J., Peng Y., Lin B., Zhu Y., Ren Z., Xiao L., Zhuang L. (2019). ACS Appl. Energy Mater..

[cit60] Zhegur A., Gjineci N., Willdorf-Cohen S., Mondal A. N., Diesendruck C. E., Gavish N., Dekel D. R. (2020). ACS Appl. Polym. Mater..

[cit61] Douglin J. C., Varcoe J., Dekel D. R. (2020). J. Power Sources Adv..

[cit62] Park E. J., Maurya S., Hibbs M. R., Fujimoto C. H., Kreuer K. D., Kim Y. S. (2019). Macromolecules.

[cit63] Chu X., Shi Y., Liu L., Huang Y., Li N. (2019). J. Mater. Chem. A.

[cit64] Maurya S., Lee A. S., Li D., Park E. J., Leonard D. P., Noh S., Bae C., Kim Y. S. (2019). J. Power Sources.

